# Dynamic networks of psychological symptoms, impairment, substance use, and social support: The evolution of psychopathology among emerging adults

**DOI:** 10.1192/j.eurpsy.2022.23

**Published:** 2022-06-13

**Authors:** Jacob J. Crouse, Nicholas Ho, Jan Scott, Richard Parker, Shin Ho Park, Baptiste Couvy-Duchesne, Brittany L. Mitchell, Enda M. Byrne, Daniel F. Hermens, Sarah E. Medland, Nicholas G. Martin, Nathan A. Gillespie, Ian B. Hickie

**Affiliations:** 1 Youth Mental Health & Technology Team, Brain and Mind Centre, University of Sydney, Sydney, New South Wales, Australia; 2 Academic Psychiatry, Institute of Neuroscience, Newcastle University, Newcastle, United Kingdom; 3 Université de Paris, Paris, France; 4 Department of Mental Health, Norwegian University of Science and Technology, Trondheim, Norway; 5 QIMR Berghofer Medical Research Institute, Brisbane, Queensland, Australia; 6 Institute for Molecular Bioscience, University of Queensland, Brisbane, Queensland, Australia; 7 Paris Brain Institute (ICM), INSERM U 1127, CNRS UMR 7225, Sorbonne University, Inria, Aramis Project-Team, 75013 Paris, France; 8 Thompson Institute, University of the Sunshine Coast, Birtinya, Queensland, Australia; 9 Virginia Institute for Psychiatric and Behavior Genetics, Virginia Commonwealth University, Richmond, Virginia, USA

**Keywords:** Cohort, network analysis, prospective, psychopathology, youth mental health

## Abstract

**Background:**

Subthreshold/attenuated syndromes are established precursors of full-threshold mood and psychotic disorders. Less is known about the individual symptoms that may precede the development of subthreshold syndromes and associated social/functional outcomes among emerging adults.

**Methods:**

We modeled two dynamic Bayesian networks (DBN) to investigate associations among self-rated phenomenology and personal/lifestyle factors (role impairment, low social support, and alcohol and substance use) across the 19Up and 25Up waves of the Brisbane Longitudinal Twin Study. We examined whether symptoms and personal/lifestyle factors at 19Up were associated with (a) themselves or different items at 25Up, and (b) onset of a depression-like, hypo-manic-like, or psychotic-like subthreshold syndrome (STS) at 25Up.

**Results:**

The first DBN identified 11 items that when endorsed at 19Up were more likely to be reendorsed at 25Up (e.g., hypersomnia, impaired concentration, impaired sleep quality) and seven items that when endorsed at 19Up were associated with different items being endorsed at 25Up (e.g., earlier fatigue and later role impairment; earlier anergia and later somatic pain). In the second DBN, no arcs met our a priori threshold for inclusion. In an exploratory model with no threshold, >20 items at 19Up were associated with progression to an STS at 25Up (with lower statistical confidence); the top five arcs were: feeling threatened by others and a later psychotic-like STS; increased activity and a later hypo-manic-like STS; and anergia, impaired sleep quality, and/or hypersomnia and a later depression-like STS.

**Conclusions:**

These probabilistic models identify symptoms and personal/lifestyle factors that might prove useful targets for indicated preventative strategies.

## Introduction

Most young people self-report psychological symptoms or social impairments at some point during the postpubertal period. For some, these are fleeting experiences, but for others, these are antecedents of major mental disorders that have a peak onset during adolescence and early adulthood (~15–30 years) [1–6]. Youth mental health research has demonstrated repeatedly that clinical high-risk states or subthreshold syndromes are often precursors of full-threshold mental disorders (e.g., depressive, bipolar, or psychotic disorders) [7–10]. However, much less research has investigated how individual symptoms recur or persist over time, covary with one another, and/or develop into clusters that precede the subthreshold syndrome, nor how social, occupational, and/or lifestyle factors (e.g., alcohol/substance use) might influence the evolution of these early manifestations of psychopathology. Improving our understanding of progression from earlier to later stages of mental illness among emerging adults is an area of major clinical, public health, and economic interest [11, 12]. Examination of these valuable questions requires access to well-characterized cohorts of young people followed prospectively, and the application of statistical models that can infer possible causal associations between many candidate variables.

Probabilistic graphical models, such as directed acyclic graphs (DAGs), are an increasingly popular approach in psychiatry for inferring possible causal associations among demographic, clinical, and biological variables measured in observational studies [13–17]. In these types of graphical models, variables are represented as “nodes” that are connected by “arcs” (or edges). Arcs specify the conditional dependencies among the variables, with the arrow of the arc pointing in the direction of a possible causal association. Dynamic Bayesian networks (DBNs) are one class of probabilistic graphical model that is increasingly used to identify potential directional associations in longitudinal data. For example, in a follow-up study of the 2000 British National Psychiatric Morbidity study, DBNs were used to identify plausible causal associations and feedback loops between depressive, anxious, and psychotic-like symptoms, as well as problems related to sleep and alcohol and substance use [18]. One advantage of DBNs is that they can be interpreted as possible causal networks linking variables across time, and have been used in time-series and longitudinal datasets to study cancer prognosis [19], gut microbiota interactions [20], gene regulatory networks [21], and functional brain connectivity [22], among other dynamic systems. Moreover, some studies have reported superiority of DBNs over traditional frequentist approaches, in that interactions between variables and outcomes are not predefined, and uncertainty in the estimations is considered [19].

The current study uses DBNs to examine the temporal relationships among self-rated psychological symptoms and associated social, occupational, and lifestyle factors across two waves of follow-up (~5–6-year interval) of a cohort of young people recruited to the Brisbane Longitudinal Twin Study (BLTS), a prospective, community-based study of adult twins and nontwin siblings. In a series of recent studies, we examined the progression of subthreshold syndromes (i.e., depression-like, hypo-manic-like, and psychotic-like) to full-threshold mood and psychotic disorders [23, 24]. Here, we focus on cohort members without evidence of a full-threshold disorder, and we model two DBNs to examine two questions: (a) what are the relationships among self-rated psychological symptoms, impairment, alcohol and other substance use, and perceived social support across the “Nineteen and Up” (19Up) and “Twenty Five and Up” (25Up) study waves; and (b) what are the relationships among these factors at 19Up and subsequent progression to a depression-like, hypo-manic-like, and/or psychotic-like subthreshold syndrome at 25Up?

## Methods

### Ethical approval, consent, and study reporting

Ethical approval for the BLTS projects was obtained from the Human Research Ethics Committee at the Queensland Institute of Medical Research (numbers: EC00278, P1212). Written informed consent was obtained for each wave from participants and their parents if applicable (i.e., participants aged <18). This study adheres to the “Strengthening the Reporting of Observational Studies in Epidemiology” (STROBE) guidelines and a checklist is provided in the Supplementary Materials.

### Study participants

Study participants are members of a prospective cohort study of twins and their nontwin siblings run at the Queensland Institute of Medical Research in Brisbane, Australia. Briefly, the BLTS began in 1992 with recruitment of twins aged ~12 years from primary and secondary schools in the greater Brisbane area, the Australian Twin Registry, and via word of mouth and media appeals in the community. Since the first wave of the BLTS at age ~12 years, cohort members have been reinvited via telephone or email to participate in follow-up waves around ages 14, 16, 19, and 25. We focus here on the “Nineteen and Up” (19Up) and “Twenty-Five and Up” (25Up) waves of the BLTS, as these individuals are in the peak age-range for onset of mental health problems. Because nontwin siblings were included and were older on average than the twins, the average age of each wave is older than would be expected based on the study names (19Up mean age = 26 years, range = 18–38; 25Up mean age = 30 years, range = 22–44) [25, 26].

### Eligibility criteria

Members of the BLTS cohort were eligible for this study based on three criteria (irrespective of relatedness, i.e., twins/siblings):

1. Had participated in both the 19Up and 25Up waves.
2. Had complete data for the variables of interest: self-rated psychological symptoms, self-rated functioning, alcohol, and other substance use (tobacco, cannabis), and perceived social support.
3. Did not meet criteria for a diagnosis of major depression, hypo/mania, and/or psychotic disorder at 19Up (according to the Composite International Diagnostic Interview; CIDI).

### Assessments

#### Individual and sociodemographic characteristics

Data about age, sex, zygosity (monozygotic, MZ; dizygotic, DZ), marital status, occupation, and highest level of education were collected using questionnaires.

#### Self-rated mental health symptoms and subthreshold syndromes

Three self-report scales were used to assess 23 psychological symptoms (see Supplementary Table S1). The 12-item Somatic and Psychological Health Report (SPHERE-12) measured the presence/absence of somatic (SOMA-6 subscale, e.g., hypersomnia, anergia) and anxious-depressive symptoms (PSYCH-6 subscale, e.g., feeling overwhelmed, hopelessness) over recent weeks [27]. Five hypo-manic symptoms (persistence >2 days of, e.g., decreased need for sleep, feeling elated) were examined using an investigator-devised self-rating scale (used in several of our studies) [23, 24, 28]. Six psychotic-like symptoms (e.g., auditory hallucinations) were assessed using a tool adapted in part from the Community Assessment of Psychic Experiences [29]. As in previous studies [30] we defined three subthreshold syndromes as follows: (a) depression-like experiences (≥3 items from the SOMA-6 subscale or ≥2 items from the PSYCH-6 subscale of the SPHERE-12); (b) hypo-manic-like experiences (cooccurrence of all five hypo-manic items); and (c) psychotic-like experiences (≥2 psychotic-like items).

#### Self-rated functioning

We estimated whether participants had any days out of role or days in bed using two modified items from the World Health Organization’s (WHO) “Disability Assessment Schedule” [31]. A response of ≥1 to the following was classified as having days out of role: “During the last few weeks how many days in total were you unable to carry out your usual daily activities fully?” Similarly, a response of ≥1 to the following was classified as having days in bed: “During the last few weeks how many days in total did you stay in bed all or most of the day because of illness or injury?”

#### Alcohol and/or substance use

Recent alcohol and/or substance use was estimated using the WHO’s “Alcohol, Smoking and Substance Involvement Screening Test” [32]. Recent tobacco or alcohol use were each defined by endorsement of “daily (or almost daily) use,” while recent cannabis use was defined by “weekly” or “daily (or almost daily) use.”

#### Perceived social support

A single item from the “Kessler Perceived Social Support” scale [33] was used to estimate whether participants perceived that they had access to a confidante: “Is there anyone in your life with whom you have a close relationship and can share your most private feelings?”

### Statistical analysis

Analyses were conducted using R (version 3.6.2) with the RStudio IDE [34] (see Supplementary Materials for details). All study variables were used as binary items in analyses. Data were collected from February 2009 to October 2018 and data analysis was performed from May to October 2020.

#### Dynamic Bayesian networks

We inferred two discrete DBNs to investigate the temporal relationships among our chosen binary variables across 19Up and 25Up following established approaches used in mental health [13, 15]. We used the *bnlearn* statistical package [35] and the hill-climbing algorithm to learn the structure of the temporal networks. Hill-climbing is a heuristic search algorithm that generates a series of random network models by adding and removing arcs (i.e., probabilistic dependencies between variables) and iterates until a goodness-of-fit score is reached (here, the Bayesian information criterion, which maximizes model fit but penalizes for complexity) [35]. Expert domain knowledge can be used to place structural priors on the inference knowledge, such that specific arcs can be forced or blocked by the learning process; however, as our approach was data-driven and hypothesis-generating, we did not force any arcs to be present in the models. For each DBN, we sampled from 1,000 bootstraps, inferred a DAG, and summarized the averaged “consensus” network of these DAGs in our main results [35]. For each DAG, we used 10 random restarts to avoid local maxima and 10 perturbing operations to randomly insert/remove arcs. Following other studies [15], we specified a threshold of 0.50 on this consensus network, so only directed arcs present in ≥50% of bootstraps are included; we also report the automatic thresholds provided by *bnlearn* for comparison. Finally, using 10-fold cross-validation, we calculate the Bayesian posterior classification error for each item at 25Up (as a measure of predictive accuracy), reporting the average loss and standard deviation of the loss over 10 runs [35].

The first DBN included all eligible participants for this study (i.e., complete data for study variables at 19Up and 25Up and no full-threshold CIDI diagnosis at 19Up) (*N* = 664). To examine progression to a subthreshold syndrome, the second DBN included only participants who did not have a subthreshold syndrome at 19Up (*N* = 538). Given the purpose of the study (i.e., exploring temporal relationships *across* 19Up–25Up), we disallowed the hill-climbing algorithm from inferring: (a) any arcs between 19Up items; (b) any arcs between 25Up items; and (c) any arcs moving backward from 25Up to 19Up. Therefore, these constraints only allowed arcs directed from 19Up items to 25Up items. For this study’s purposes, all eligible participants are treated as singletons (i.e., separate DBNs were not modeled within MZ/DZ pairs); however, a twin/nontwin item was included in the DBNs.

## Results

### Sample characteristics

As of March 2020, data were available for 2,773 individuals who participated in 19Up and 2,627 individuals who participated in 25Up. Of these, 2,292 participated in both waves, and a total of 664 participants met all eligibility criteria for this study (i.e., participants with no missing data for our variables of interest and no evidence of a CIDI diagnosis at 19Up).

As shown in Table 1, ~60% of the study sample were female (*N* = 402); the median age at the 19Up wave was 26 years (IQR = 23–29) and at the 25Up wave was 30 years (IQR = 27–34). Over 99% of participants were in the 18–35 age-range at 19Up and 88% also were at 25Up. There were 224 MZ individuals (56 complete MZ pairs), 230 DZ individuals (29 complete DZ pairs), and 210 nontwin siblings. While the age and sex distributions of our sample are very similar to the total 19Up and 25Up samples, our distribution of complete twin pairs is comparatively lower, probably due to our requirement for complete data and differential completion of waves within twin pairs.

**Table 1.** Sociodemographic characteristics of the final sample at 19Up and 25Up (*N* = 664).

	*N* (%) or M (SD)	
	19Up	25Up
Age, years	26.2 (4.0)	30.3 (4.4)
Sex (female)	402 (60.5)	402 (60.5)
Marital status		
Married	187 (28.2)	452 (68.1)
Separated, divorced, widowed	9 (1.4)	75 (11.3)
Never married	468 (70.5)	137 (20.6)
Primary occupation		
Full-time	438 (66.0)	460 (69.3)
Part-time	82 (12.4)	78 (11.7)
Studying	85 (12.4)	32 (4.8)
Home duties	31 (4.7)	49 (7.4)
Employed, not working (e.g., illness)	10 (1.5)	8 (1.2)
Receiving sickness/disability benefits	3 (0.5)	2 (0.3)
Volunteer	5 (0.8)	2 (0.3)
Unemployed	9 (1.4)	5 (0.8)
Education (highest level)		
Postgraduate degree	105 (15.8)	146 (22.0)
Undergraduate degree	324 (48.8)	304 (45.8)
Certificate/diploma	158 (23.8)	160 (24.1)
Junior/senior high school	77 (11.6)	52 (7.8)

Table 2 reports the prevalence rates for the study items across 19Up and 25Up (i.e., symptoms, impairment). A total of 126 individuals met criteria for at least one subthreshold syndrome at 19Up. Of the remaining 538 individuals, 43 progressed to a depression-like subthreshold syndrome by 25Up, 61 progressed to a hypo-manic-like subthreshold syndrome by 25Up, and 13 progressed to a psychotic-like subthreshold syndrome by 25Up.

**Table 2.** Prevalence rates of self-rated symptoms, subthreshold syndromes, impairment, substance use, and perceived social support at 19Up and 25Up (*N* = 664).

	Prevalence (%)	
	19Up	25Up
Self-rated symptoms		
Feeling nervous or tense	16.9	14.9
Feeling unhappy or depressed	10.4	10.7
Feeling stressed	22.6	15.8
Feeling overwhelmed	18.5	20.0
Lost confidence	12.5	12.7
Hopelessness	7.7	5.7
Somatic pain	20.3	20.0
Hypersomnia	44.9	47.6
Fatigue	15.4	12.3
Impaired sleep quality	31.6	35.8
Impaired concentration	17.3	11.3
Anergia	24.1	16.4
Feeling elated	38.7	39.2
Increased self-esteem or self-confidence	30.7	35.5
Reduced need for sleep	16.4	20.0
Increased pressure of sleep	19.1	22.6
Increased physical activity	28.5	32.1
Thoughts not your own	4.1	3.8
Third party auditory hallucinations	0.8	0.6
Heard voices (when alone)	1.2	2.0
Feeling threatened by others	1.4	2.1
People are against me	2.1	3.0
Thought withdrawal	0.2	0.8
Subthreshold syndromes		
DLE	11.3	11.0
HMLE	8.0	13.7
PLE	1.5	3.0
Impairment		
Any days out of role (past month)	19.7	20.0
Any days in bed (past month)	15.1	15.2
Substance use		
Daily (or almost daily) tobacco	6.5	4.2
Daily (or almost daily) alcohol	8.6	1.1
Weekly or more frequent cannabis	1.7	1.7
Perceived social support		
Does not have a confidante	9.2	5.1

Abbreviations: DLE, depression-like experience; HMLE, hypo-manic-like experience; PLE, psychosis-like experience.

### Associations among mental health symptoms, substance use, social support, and impairment

**Figure 1.**
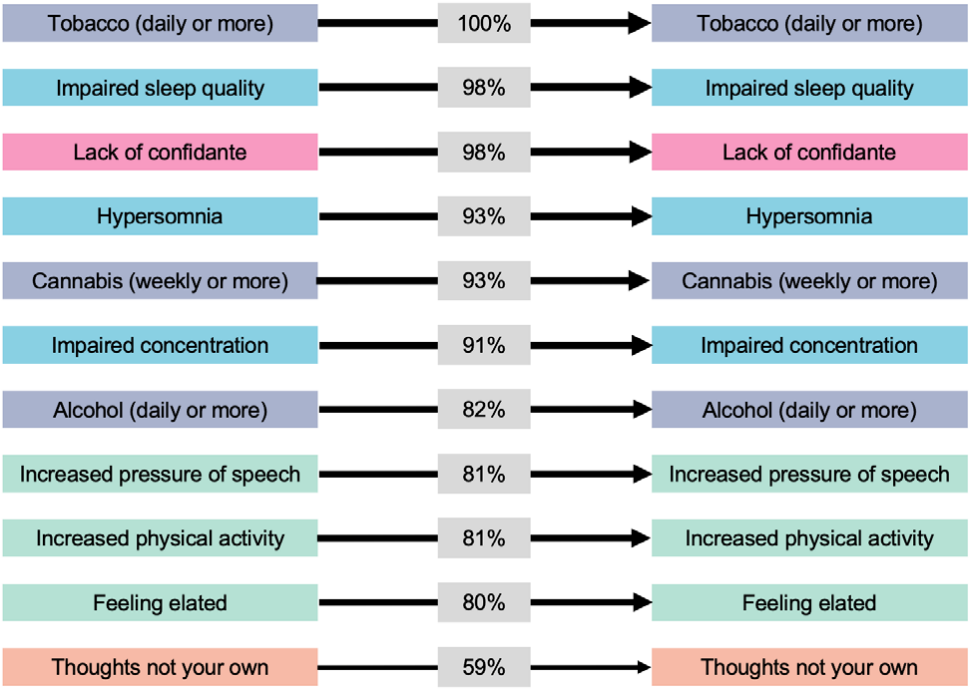
Within-item relationships among symptoms, impairment, substance use, and perceived social support from the first dynamic Bayesian network. Only arcs present in ≥50% of 1,000 bootstraps are displayed. Line thickness and percentages represent the proportion of bootstraps each arc was present in. Colors represent domains (blue, anxious-depressive; green, hypo-manic; orange, psychotic-like; purple, substance use; and pink, social support).

**Figure 2.**
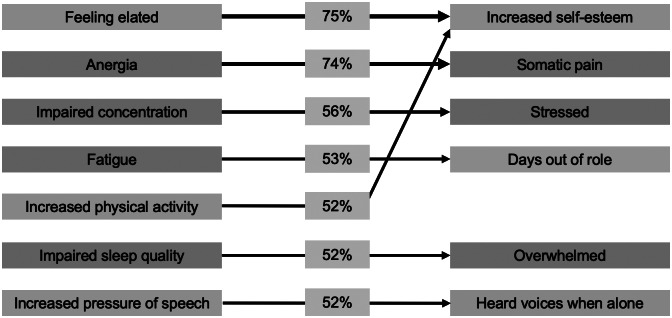
Cross-item relationships among self-rated symptoms and impairment from the first dynamic Bayesian network. Only arcs present in ≥50% of 1,000 bootstraps are displayed. Line thickness and percentages represent the proportion of bootstraps each arc was present in. Colors represent domains (blue, anxious-depressive; green, hypo-manic; orange, psychotic-like; and pink, social support).

The findings of the first DBN are shown in Figure 1 (within-item arcs) and Figure 2 (cross-item arcs). There was a close match between our a priori threshold of 0.50 and the automatic threshold provided by *bnlearn* (0.49). Effect sizes for the marginal associations between these items at 19Up on items at 25Up are presented in Supplementary Tables S2 and S3, and the posterior classification errors for each node at 25Up are presented in Supplementary Table S5.

As shown in Figure 1, there were a total of 11 items (seven of which were self-rated psychological symptoms) that if endorsed at the 19Up wave were associated with an increased probability of also being endorsed by the same individual at 25Up. In order of statistical confidence in the robustness of each arc (as a % of bootstrap estimations) the within-item arcs included: daily (or almost daily) tobacco use (100%), lack of a confidante (98%), impaired sleep quality (98%), weekly or more frequent cannabis use (93%), impaired concentration (91%), daily (or almost daily) alcohol use (82%), increased pressure of speech (81%), increased physical activity (81%), feeling elated (80%), and “thoughts not your own” (59%).

As shown in Figure 2, there were seven items (all psychological symptoms) that if endorsed at the 19Up wave were associated with an increased probability of a *different* item being endorsed at 25Up. In order of confidence in the robustness of each arc, these cross-item arcs included: feeling elated at 19Up and increased self-esteem at 25Up (75% bootstrap estimations), anergia at 19Up and somatic pain at 25Up (74%), impaired concentration at 19Up and feeling stressed at 25Up (56%), fatigue at 19Up and having days out of role at 25Up (53%), impaired sleep quality at 19Up and feeling overwhelmed at 25Up (52%), increased psychomotor speed (speech) at 19Up and heard voices when alone at 25Up (52%), and increased activity (physical) at 19Up and increased self-esteem at 25Up (52%).

### Progression to a subthreshold syndrome from 19Up to 25Up

**Figure 3.**
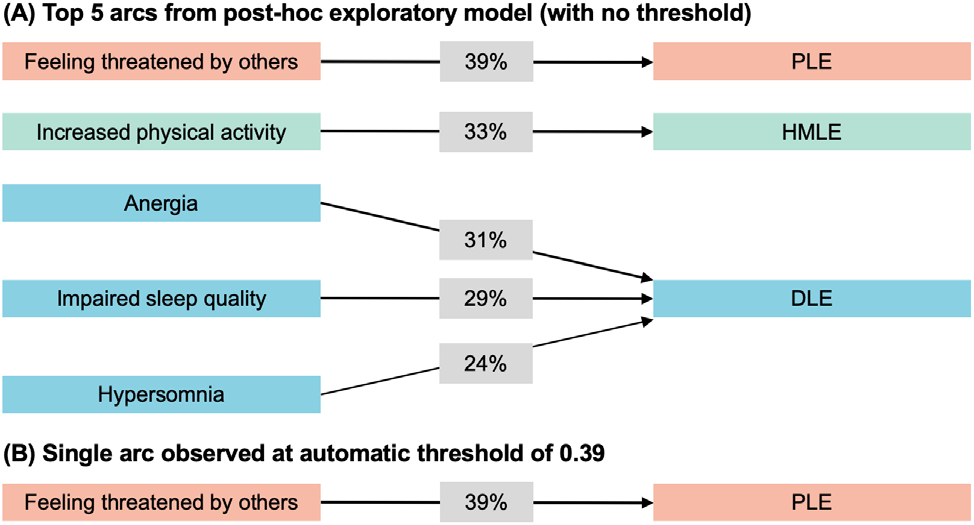
Self-rated symptoms at 19Up associated with progression to a subthreshold syndrome at 25Up from the second Dynamic Bayesian network. (A) Because no arcs were observed at our a priori threshold of 50% bootstrap estimations (0.50), we present a post hoc exploratory model with no threshold; only the top five most certain arcs are shown. (B) Here, we present the single arc that was observed at the automatic detection threshold calculated by *bnlearn* (0.39). Line thickness and percentage represent the proportion of bootstraps each arc was observed in. Colors represent domains (blue, anxious-depressive; green, hypo-manic; and orange, psychotic-like). DLE, depression-like experience; HMLE, hypo-manic-like experience; PLE, psychosis-like experience.

Using our a priori threshold of 0.50 for arc presence across the 1,000 bootstraps, no arcs were suitable for inclusion in our final “consensus” model (as no arcs were observed in ≥50% of the bootstraps). Subsequently, we conducted a fully exploratory post hoc analysis, removing the 0.50 threshold. In Figure 3, we summarize the findings of this exploratory analysis, reporting only the top five most certain arcs observed across bootstrap estimations (which should be treated with caution). Effect sizes for the marginal associations between these items at 19Up on items at 25Up are presented in Supplementary Table S4, and the posterior classification errors for each node at 25Up are presented in Supplementary Table S6. Notably, only the arc from “feeling threatened by others” at 19Up to a psychotic-like STS at 25Up was above the automatic threshold provided by *bnlearn* (automatic threshold = 0.39).

## Discussion

### Primary findings

We used probabilistic graphical models to explore how psychological symptoms, impairment, social support, and substance use interrelate across early adulthood, and how they increase the probability of progression to subthreshold mood or psychotic syndromes. We focus this discussion primarily on the finding that symptoms from the anxious-depressive and hypo-manic dimensions were most consistently related to themselves and the presence of other symptoms longitudinally. We also note that our post hoc exploratory model identified putative precursor symptoms of subthreshold syndromes, and these findings will be briefly considered alongside the study’s limitations.

The first DBN revealed that young adults who endorsed one or more of a set of 11 symptoms and factors at 19Up were more likely to endorse these symptoms again ~5–6 years later. Furthermore, seven specific symptoms were found to have cross-item arcs, indicating that their presence at 19Up increased the probability of *other* symptoms being present at 25Up. Of note, most of the within-item arcs and all the cross-item arcs had a root node at 19Up in the anxious-depressive or hypo-manic dimensions. While we could discuss several findings, we focus on two main points which also shed light on the second DBN. First, several symptoms at 19Up from the anxious-depressive dimension—namely impaired sleep quality and impaired concentration—were associated with a higher probability of their persistence and presence of other symptoms at 25Up. Second, several symptoms from the hypo-manic dimension—namely feeling elated, increased physical activity, and increased pressure of speech—were associated with their own persistence and presence of other items from 25Up, both *homotypically* (e.g., increased self-esteem) and *heterotypically* (e.g., heard voices when alone). Whether these within-and cross-item relationships reflect shared heritability of symptom types/dimensions [36], common lifestyle factors (e.g., sleep–wake disturbance) [37, 38], a positive response style, or a complex causal network in which symptoms activate/maintain each other [39] are important questions with different etiologic and treatment implications.

In the second DBN, no arcs met our a priori cut-off for inclusion in the “consensus” model (threshold = 0.50), and we accordingly only discuss the top five most certain arcs from our exploratory post hoc analysis that were associated with progression from an asymptomatic or nonspecific symptom state to one of three subthreshold syndromes (these findings should be treated with caution). First, feeling threatened by others at 19Up was associated with progression to a psychotic-like subthreshold syndrome, consistent with reports from the USA [7, 40] and China [41] showing that higher levels of suspiciousness predict conversion from a clinical high risk state to a full-threshold psychotic disorder; it is worth noting that this was the only arc in the second DBN above the automatic threshold provided by *bnlearn* (threshold = 0.39). Second, increased physical activity at 19Up was associated with an increased likelihood of progression to a hypo-manic-like subthreshold syndrome. This finding is consistent with a recent network analysis at a single timepoint in BLTS showing that increased physical activity was an influential node among youth with recent-onset bipolar disorder [23]. Another study showed that subsyndromal manic symptoms were associated with a new-onset bipolar spectrum disorder among youth at familial risk of bipolar disorder [42]. Finally, anergia, impaired sleep quality, and hypersomnia were associated with a higher likelihood of progression to a depressive-like subthreshold syndrome, consistent with evidence that sleep disturbances are associated with increased risk of first onset of depression in young people [43]. While these findings suggest some potential precursors of subthreshold syndromes, they should be considered preliminary considering the exploratory nature of the analysis.

### Limitations

This study has several important limitations. First, our analytic approach was data-driven, and we did not specify hypotheses about expected arcs; accordingly, these findings need confirmation. Second, while the inclusion of older nontwin siblings inflated the average age of the sample, most participants are in the peak age-range for onset of significant mental health problems (88% were aged 18–30 years at 19Up, 50% were aged 18–30 at 25Up). Relatedly, other epidemiologic studies, such as the National Comorbidity Survey Replication [44], have reported that around half of respondents dated the onset of their mental disorder before age 14, while in the current study, formal interview-based diagnoses were recorded at the 19Up wave; accordingly, it is possible that BLTS differs somewhat from other epidemiologic samples. Third, the interval between waves was ~5–6 years, and it is probable that meaningful relationships occurring at shorter timescales were missed. Fourth, the repeated cross-sectional design means that we cannot confirm persistence of symptoms over time. Fifth, we selected social/lifestyle factors measured using simple assessments, sometimes employing single items to represent complex phenomena (e.g., social support/having a confidante), and we did not include biological data (e.g., genetic risk). Sixth, while we included a twin status item in the DBNs (which was not influential over any other nodes), we did not stratify the analyses to better leverage the familial structure of the data. Finally, there are two important points regarding generalizability. Sample attrition was significant, with ~25% of the total sample eligible for this study; ≈1,000 participants were deemed ineligible because they were not administered the SPHERE at 19Up, and of the remaining participants, 346 were excluded because they already met criteria for a full-threshold mood and/or psychotic disorder by 19Up. For a discussion of the impacts of these types of biases (e.g., attrition) on DBNs, we encourage interested readers to consult detailed resources [45]. Relatedly, participants were drawn from the community, and it is unclear whether the findings are generalizable to other population-based youth cohorts or to young people in the early phases of mental disorders accessing clinical services.

## Conclusions and Future Directions

Two key strengths of our methodologic approach are that DBNs can be interpreted as causal networks that give rise to novel hypothesis for future study, and second, that they consider the degree of uncertainty in the data, which can help researchers decide which hypotheses may be most fruitful to test. Our models give rise to the hypotheses that clinical or public health strategies that target a specific set of symptoms—including impaired sleep quality, impaired concentration, and increased activity, among others of varying degrees of confidence—may reduce the likelihood that these same symptoms and others would be observed again in the same individuals. External studies are needed to confirm whether these symptoms are reliably associated with persistence and emergence of other symptoms and/or progression to more severe and impairing illnesses.

## Supplementary material

For supplementary material accompanying this paper visit http://doi.org/10.1192/j.eurpsy.2022.23. click here to view supplementary material
