# Supplementary material for: Dynamic networks of psychological symptoms, impairment, substance use, and social support: The evolution of psychopathology among emerging adults
Source: Eur Psychiatry. 2022 Jun 13;65(1):e32. doi: 10.1192/j.eurpsy.2022.23 (PMC9280922; doi:10.1192/j.eurpsy.2022.23)
Supplement: Supplementary file 1 [file epasup.zip › S0924933822000232sup002.docx]

**Supplementary Table 1. Item names and questions for three self-rated mental health screening tools.**

| **Item name (short)** | **Item question** |
| --- | --- |
| **SPHERE-12 – “Anxious-depressive”** | |
| *Over the past few weeks have you been troubled by:* | |
| Feeling nervous or tense | Feeling nervous or tense |
| Feeling unhappy or depressed | Feeling unhappy and depressed |
| Feeling stressed | Feeling constantly under strain |
| Feeling overwhelmed | Everything getting on top of you |
| Lost confidence | Losing confidence |
| Hopelessness | Being unable to overcome difficulties |
| **SPHERE-12 – “Somatic”** | |
| *Over the past few weeks have you been troubled by:* | |
| Somatic pain | Muscle pain after activity |
| Hypersomnia | Needing to sleep longer |
| Fatigue | Prolonged tiredness after activity |
| Impaired sleep quality | Poor sleep |
| Impaired concentration | Poor concentration |
| Anergia | Tired muscles after activity |
| **Psychotic-like symptom screener** | |
| *Have you ever...* | |
| Thoughts not your own | Felt as if the thoughts in your head were not your own? |
| Third party auditory hallucinations | Heard voices talking to each other when you were alone? |
| Heard voices (when alone) | Heard voices when you were alone? |
| Paranoia | Felt that many people around you might hurt or harm you in some way? |
| People are against me | Felt as if many people around you were plotting against you? |
| Thought withdrawal | Felt as if the thoughts in your head are being taken away from you? |
| **Hypo-manic symptom screener** | |
| *Have you ever experienced a definite period where for more than two (2) or three (3) days:* | |
| Feeling elated | You felt much happier or more cheerful than usual? |
| Increased self-esteem or self-confidence | You felt much more self-confident than usual? |
| Reduced need for sleep | You needed much less sleep than usual? |
| Increased pressure of sleep | You talked much more than usual? |
| Increased physical activity | You were much more active than usual? |

**Supplementary Table 2. Associations between items at 19Up and 25Up included in the first “consensus” Dynamic Bayesian Network.** Only “within-item” arcs are presented in this table.

| **19Up Item and 25Up Item** | **Pearson’s**  **correlation (r)** | **P-value** | **Pearson’s product-moment**  **correlation coefficient (r)** | **P-value** |
| --- | --- | --- | --- | --- |
| Tobacco (19Up) 🡪  Tobacco (25Up) | 0.584 | <0.001 | 0.571 | <0.001 |
| Impaired sleep quality (19Up) 🡪  Impaired sleep quality (25Up) | 0.282 | <0.001 | 0.215 | <0.001 |
| Lack of confidante (19Up) 🡪  Lack of confidante (25Up) | 0.130 | <0.001 | 0.062 | 0.120 |
| Hypersomnia (19Up) 🡪  Hypersomnia (25Up) | 0.268 | <0.001 | 0.178 | <0.001 |
| Cannabis (19Up) 🡪  Cannabis (25Up) | 0.538 | <0.001 | 0.537 | <0.001 |
| Impaired concentration (19Up) 🡪  Impaired concentration (25Up) | 0.264 | <0.001 | 0.195 | <0.001 |
| Alcohol (19Up) 🡪  Alcohol (25Up) | 0.232 | <0.001 | 0.213 | <0.001 |
| Increased pressure of speech (19Up) 🡪  Increased pressure of speech (25Up) | 0.305 | <0.001 | 0.165 | <0.001 |
| Increased physical activity (19Up) 🡪  Increased physical activity (25Up) | 0.303 | <0.001 | 0.139 | <0.001 |
| Feeling elated (19Up) 🡪  Feeling elated (25Up) | 0.268 | <0.001 | 0.151 | <0.001 |
| Thoughts not your own (19Up) 🡪  Thoughts not your own (25Up) | 0.200 | <0.001 | 0.175 | <0.001 |

**Supplementary Table 3. Associations between items at 19Up and 25Up included in the first “consensus” Dynamic Bayesian Network.** Only “cross-item” arcs are presented in this table.

| **19Up Item and 25Up Item** | **Pearson’s**  **correlation (r)** | **P-value** | **Pearson’s product-moment**  **correlation coefficient (r)** | **P-value** |
| --- | --- | --- | --- | --- |
| Feeling elated (19Up) 🡪  Increased self-esteem (25Up) | 0.288 | <0.001 | 0.123 | 0.002 |
| Anergia (19Up) 🡪  Somatic pain (25Up) | 0.255 | <0.001 | 0.119 | 0.003 |
| Impaired concentration (19Up) 🡪  Stressed (25Up) | 0.194 | <0.001 | 0.093 | 0.019 |
| Fatigue (19Up) 🡪  Days out of role (25Up) | 0.173 | <0.001 | 0.115 | 0.004 |
| Increased physical activity (19Up) 🡪  Increased self-esteem (25Up) | 0.278 | <0.001 | 0.106 | 0.008 |
| Impaired sleep quality (19Up) 🡪  Overwhelmed (25Up) | 0.186 | <0.001 | 0.103 | 0.010 |
| Increased pressure of speech (19Up) 🡪  Heard voices when alone (25Up) | 0.152 | <0.001 | 0.137 | <0.001 |

**Supplementary Table 4. Associations between items at 19Up and 25Up included in the second “consensus” Dynamic Bayesian Network.**

| **19Up Item and 25Up Item** | **Pearson’s**  **correlation (r)** | **P-value** | **Pearson’s product-moment**  **correlation coefficient (r)**^a^ | **P-value** |
| --- | --- | --- | --- | --- |
| Feeling threatened by others (19Up) 🡪  Psychotic-like subthreshold syndrome (25Up) | 0.313 | <0.001 | — | — |
| Increased physical activity (19Up) 🡪  Hypo/manic-like subthreshold syndrome (25Up) | 0.154 | <0.001 | — | — |
| Anergia (19Up) 🡪  Depression-like subthreshold syndrome (25Up) | 0.131 | <0.001 |  |  |
| Impaired sleep quality (19Up) 🡪  Depression-like subthreshold syndrome (25Up) | 0.123 | 0.004 | — | — |
| Hypersomnia (19Up) 🡪  Depression-like subthreshold syndrome (25Up) | 0.115 | 0.008 | — | — |

**Note:** ^a^ Pearson’s product-moment correlation coefficients were not calculated because the subthreshold syndromes at 25Up are composed of symptom items at 19Up.

**Supplementary Table 5. Posterior classification errors for variables at 25Up in first dynamic Bayesian network (using 10-fold cross-validation over 10 runs).**

| **Target variable at 25Up** | **Average loss**  **over 10 runs** | **Standard deviation**  **of the loss** |
| --- | --- | --- |
| Feeling nervous or tense | 0.172 | 0.009 |
| Feeling unhappy or depressed | 0.128 | 0.004 |
| Feeling stressed | 0.166 | 0.004 |
| Feeling overwhelmed | 0.170 | 0.007 |
| Lost confidence | 0.129 | 0.003 |
| Hopelessness | 0.087 | 0.004 |
| Somatic pain | 0.141 | 0.001 |
| Hypersomnia | 0.237 | 0.005 |
| Fatigue | 0.154 | 0.003 |
| Impaired sleep quality | 0.255 | 0.007 |
| Impaired concentration | 0.155 | 0.004 |
| Anergia | 0.138 | 0.005 |
| Feeling elated | 0.089 | 0.003 |
| Increased self-esteem | 0.089 | 0.002 |
| Reduced need for sleep | 0.151 | 0.004 |
| Increased pressure of sleep | 0.111 | 0.007 |
| Increased physical activity | 0.135 | 0.005 |
| Thoughts not your own | 0.052 | 0.003 |
| Third party auditory hallucinations | 0.010 | 0.002 |
| Heard voices (when alone) | 0.026 | 0.002 |
| Feeling threatened by others | 0.041 | 0.003 |
| People are against me | 0.050 | 0.004 |
| Thought withdrawal | 0.013 | 0.001 |
| Daily (or almost daily) tobacco | 0.061 | 0.007 |
| Daily (or almost daily) alcohol | 0.012 | < 0.001 |
| Weekly or more frequent cannabis | 0.033 | 0.002 |

**Supplementary Table 6. Posterior classification errors for variables at 25Up in second dynamic Bayesian network (using 10-fold cross-validation over 10 runs).**

| **Target variable at 25Up** | **Average loss**  **over 10 runs** | **Standard deviation**  **of the loss** |
| --- | --- | --- |
| Depression-like subthreshold syndrome | 0.080 | 0.001 |
| Hypo-mania-like subthreshold syndrome | 0.114 | 0.003 |
| Psychotic-like subthreshold syndrome | 0.021 | 0.001 |

**Supplementary Methods**

*Computing*

Following a caution by McNally and colleagues (McNally *et al.*, 2017) that different computer operating systems and software versions may generate different DAGs, we report that our analysis was completed on a MacBook Pro running macOS Catalina (version 10.15.7; CPU = 2.4 GHz 8-Core Intel Core i9, RAM = 64 GB 2667 MHz DDR4) and *bnlearn* package version 4.5.

**REFERENCES**

**McNally, R. J., Heeren, A. & Robinaugh, D. J.** (2017). A Bayesian network analysis of posttraumatic stress disorder symptoms in adults reporting childhood sexual abuse. *Eur J Psychotraumatol* **8**, 1341276.
